# Low voltage-driven oxide phototransistors with fast recovery, high signal-to-noise ratio, and high responsivity fabricated via a simple defect-generating process

**DOI:** 10.1038/srep31991

**Published:** 2016-08-24

**Authors:** Myeong Gu Yun, Ye Kyun Kim, Cheol Hyoun Ahn, Sung Woon Cho, Won Jun Kang, Hyung Koun Cho, Yong-Hoon Kim

**Affiliations:** 1 School of Advanced Materials Science and Engineering, Sungkyunkwan University, 2066 Seobu-ro, Jangan-gu, Suwon, Gyeonggi-do, 16419, Republic of Korea; 2 SKKU Advanced Institute of Nanotechnology (SAINT), Sungkyunkwan University, 2066 Seobu-to, Jangan-gu, Suwon, Gyeonggi-do, 16419, Republic of Korea

## Abstract

We have demonstrated that photo-thin film transistors (photo-TFTs) fabricated via a simple defect-generating process could achieve fast recovery, a high signal to noise (S/N) ratio, and high sensitivity. The photo-TFTs are inverted-staggered bottom-gate type indium-gallium-zinc-oxide (IGZO) TFTs fabricated using atomic layer deposition (ALD)-derived Al_2_O_3_ gate insulators. The surfaces of the Al_2_O_3_ gate insulators are damaged by ion bombardment during the deposition of the IGZO channel layers by sputtering and the damage results in the hysteresis behavior of the photo-TFTs. The hysteresis loops broaden as the deposition power density increases. This implies that we can easily control the amount of the interface trap sites and/or trap sites in the gate insulator near the interface. The photo-TFTs with large hysteresis-related defects have high S/N ratio and fast recovery in spite of the low operation voltages including a drain voltage of 1 V, positive gate bias pulse voltage of 3 V, and gate voltage pulse width of 3 V (0 to 3 V). In addition, through the hysteresis-related defect-generating process, we have achieved a high responsivity since the bulk defects that can be photo-excited and eject electrons also increase with increasing deposition power density.

Photodetectors used to electrically detect light can be typically classified into three categories based on the device structure: photoresistors^1^,^2^,^3^, photodiodes^4^,^5^,^6^, and phototransistors^7^,^8^,^9^,^10^,^11^,^12^,^13^,^14^,^15^,^16^. Among them, phototransistors have impressive advantages such as high responsivity and a high signal to noise (S/N) ratio^17^. In particular, photo-thin film transistors (photo-TFTs)^13^,^14^,^15^,^16^ are more attractive than other types of phototransistors such as bipolar junction phototransistors^7^,^8^,^9^ and complementary metal-oxide-semiconductors^10^,^11^,^12^ because they are simply and inexpensively integrated as an active matrix photosensor array. In view of sensing materials, oxide semiconductors are more suitable for photo-TFTs than conventional materials including amorphous silicon (*a*-Si) due to their high sensing speed, high responsivity, and high S/N ratio^13^. Moreover, direct detection of the wavelength of interest can be realized in oxide-based photo-TFTs by measuring different photocurrent levels at different wavelengths. On the other hand, Si-based photo-TFTs have no wavelength selectivity due to their nearly constant responsivity over a broad range of wavelengths (400–750 nm)^13^. Thus, color filters for sensing a certain wavelength are necessary in Si-based photosensor arrays. The wavelength selectivity of oxide-based photo-TFTs is attributed to two facts: i) photo-excitation of subgap states and the subsequent liberation of electrons, and ii) an increment of the amount of the photo-excited subgap states and electrons along with decreasing wavelength.

However, the photo-excitation of subgap states such as ionized oxygen vacancies in oxide-based photo-TFTs is accompanied by lattice relaxation and results in a persistent photocurrent (PPC) even after turning the light off^13^,^14^. This leads to long-term recovery of oxide-based photo-TFTs. Thus, in order to solve the PPC problem, dual gate- or positive gate bias pulse voltage (PBP)-driven photo-TFTs were proposed in previous reports^13^,^14^,^15^,^16^. In dual gate-driven photo-TFTs proposed by Hsieh *et al.*^15^ the subthreshold photo-leakage current flows through the top-gate operated channel under light illumination because of source barrier lowering due to the photo-generated hole accumulation at the source side. Once the light is switched off, recombination between electrons and holes occurs and the photo-leakage current vanishes. Meanwhile, in PBP-driven photo-TFTs demonstrated by Ahn and Jeon *et al.*^13^,^14^ the PPC was erased by applying a PBP of 5 or 10 V for 10 ns or 1 *μ*s. The removal of the PPC by the PBP was ascribed only to PBP-accelerated recombination events between oxygen vacancies and excess electrons in the bulk semiconductor since the PPC dominantly originated from the bulk IZO layers in IGZO/IZO or IGZO/IZO/IGZO multi-channel layers.

However, these PPC-free photo-TFTs adopted additional processes for fabricating dual gate or multi-channel structures. Moreover, the subthreshold photo-leakage current (a kind of signal) of the dual gate-driven photo-TFTs is lower than 0.1 nA and the PBP-driven photo-TFTs were operated at a wide gate voltage (*V*_G_) pulse width of 10 V (−5 to 5 V) or 17 V (−7 to 10 V). Chen *et al*. also proposed the PBP-driven PPC-free photo-TFTs with unannealed IGZO single-channel layers, which were operated at a wide *V*_G_ pulse width of 10 V (0 to 10 V)^16^. They used a large amount of natural defects within the unannealed IGZO or at the IGZO/gate insulator interface that act as electron traps to erase the PPC^16^. However, the unannealed IGZO films is generally of low quality and have inhomogeneous distribution of defects resulting in poor uniformity even in a small area of 1 cm^2 ^18. In the fabrication of photosensor arrays, post-thermal processes such as passivation or annealing are necessary for defect homogenization^13^,^14^,^15^. Therefore, the fabrication of PPC-free photo-TFTs with simple- and uniform-channel layers is an issue to have to be solved.

In this study, photo-TFTs were fabricated via a simple defect-generating process to solve the PPC problem by using artificially-controlled hysteresis-related defects in combination with recombination events. The defect-generating process has three key points: i) adoption of the bottom-gate type TFT structure with annealed IGZO, ii) use of atomic layer deposition (ALD)-derived Al_2_O_3_ gate insulators, and iii) artificial control of hysteresis-related defects via control of deposition power density. The hysteresis-related defects as well as bulk defects created via the process lead to the photo-TFTs possessing fast recovery, a low light-off state current (high S/N ratio), and high sensitivity even at low operation voltages including the photocurrent-sensing voltages (*V*_G_ = 0 V and *V*_D_ = 1 V) and the PPC-erasing voltage (PBP = 3 V).

## Results

### Simple structure and easy fabrication process to enlarge hysteresis-related defects for fast recovery: Bottom gate IGZO TFTs with ALD-derived Al_2_O_3_ gate insulators

**Figure 1:**
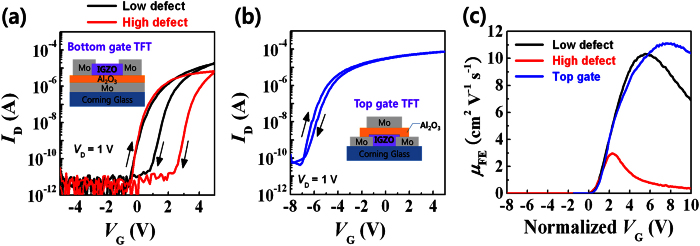
(**a**) Hysteresis characteristics of the bottom-gate TFTs using IGZO channel layers with different deposition power densities of 0.62 (low defect) and 1.85 W cm^−2^ (high defect). (**b**) Hysteresis characteristics of the top-gate TFT using IGZO channel layers with a deposition power density of 1.85 W cm^−2^. (**c**) Field-effect mobility (*μ*_FE_) vs. normalized *V*_G_ plots of all of the IGZO TFTs.

Figure 1a shows the transfer curves of the IGZO TFTs on 100 nm thick Al_2_O_3_ gate insulators deposited by an atomic layer deposition (ALD) process at 150 °C. The IGZO channel layers were deposited by RF magnetron sputtering with different deposition power densities of 0.62 and 1.85 W cm^−2^. The schematic view of the TFT structure is illustrated in the inset of Fig. 1a which corresponds to an inverted-staggered bottom-gate structure. For the measurements of the transfer characteristics, at a fixed drain voltage (*V*_D_) of 1 V, the gate voltage (*V*_G_) was swept forward from −5 to 5 V and reversely from 5 to −5 V. The threshold voltage (*V*_th_) was defined as the gate voltage at which a drain current (*I*_D_) of 10 × L/W nA (=1 nA) flows (constant drain current method) and the hysteresis value (Δ*V*_Hys_) is the threshold voltage shift in the hysteresis loop. As seen in Fig. 1a, the IGZO TFT obtained with a low deposition power density of 0.62 W cm^−2^ (hereafter, referred to as the low defect TFT) had a relatively small Δ*V*_Hys_ of 1.2 V. On the other hand, the IGZO TFT fabricated with a high deposition power density of 1.85 W cm^−2^ (hereafter, referred to as the high defect TFT) exhibited a large Δ*V*_Hys_ of 2.9 V. This dependency of the Δ*V*_Hys_ on the deposition power density may originate from process damage on the surface of the Al_2_O_3_ insulator due to ion bombardment during the IGZO deposition. To confirm whether there is plasma damage during the deposition of IGZO, we fabricated a plasma damage-free IGZO TFT with a top-gate structure, as illustrated in the inset of Fig. 1b. When fabricating the top-gate TFT, the IGZO channel layer was deposited with a high deposition power density of 1.85 W cm^−2^ and then, the Al_2_O_3_ insulator was also deposited by the same ALD process. Thus, the plasma damage on the surface of the Al_2_O_3_ insulator by the ion bombardment during IGZO deposition does not need to be considered in the top-gate TFT. Consequently, unlike the two bottom-gate TFTs (both low and high defect TFTs), the top-gate TFT had a very small Δ*V*_Hys_ of 0.3 V in spite of a broader sweep range from −8 to 5 V. Thus, we can conclude that the damage on the Al_2_O_3_ insulator (defect creation in the bulk Al_2_O_3_ insulator near the channel layer/insulator interface) by the ion bombardment is the culprit of the Δ*V*_Hys_ in both the low and high defect TFTs. It is well known that there are three origins of Δ*V*_Hys_ in TFTs: i) channel layer/insulator interface-induced effect, ii) residual dipole-induced effect (i.e. slow polarization in the bulk insulator), and iii) effects of charges injected from the gate electrode^19^. Hydrogen–related impurity can be also considered as a cause of hysteresis in oxide-based TFTs, since hydrogen diffusion is much faster in amorphous oxide matrix^20^,^21^. The Al_2_O_3_ films deposited by ALD can be contain large amounts of hydrogens due to insufficient chemical reaction between precursors at low deposition temperatures. However, since the growth temperature of the Al_2_O_3_ layers did not significantly affect the Δ*V*_Hys_ (Supplementary Figure S5), the hydrogen-related impurities in our photo-TFTs can be ignored. Based on the results of the top-gate TFT, it is clear that in the bottom-gate TFTs, both slow polarization and charge injection from the gate electrode to bulk insulator are not significant but the channel layer/insulator interface-induced effect, i.e. charge trapping/injection near the channel-layer/insulator interface, is dominant. The density of the hysteresis-related defects (*N*_T_) is proportional to Δ*V*_Hys_ and can be estimated by the following equation^22^:


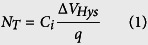


where *C*_i_, and *q* are the gate insulator capacitance per unit area and the elementary charge, respectively. *C*_i_ was estimated to be 7.97 × 10^−8^ F cm^−2^ based on the dielectric constant of Al_2_O_3_ (9.0). The estimated *N*_T_ values for the TFTs with different deposition power density and with different TFT structures are listed in Supplementary Table S1. There are four general types of charges that can play the role of the trap sites inducing the hysteresis: i) interface trap charge at the channel layer/insulator interface, ii) fixed oxide charge near the interface, iii) oxide trapped charge within the gate insulator, and vi) mobile ionic charge within the gate insulator^23^. Among them, both the oxide trapped charges and the mobile ionic charges within the gate oxide can be ignored because the top-gate TFT did not show any significant hysteresis. Thus, the large hysteresis in the bottom-gate TFTs is related with the interface trapped charge and the fixed oxide charge at/near the channel layer/insulator interface, and these might be generated by the ion bombardment during the deposition of the IGZO channel layers. To investigate the defect (deposition power density) dependency of the field-effect mobility (*μ*_FE_), as shown in Fig. 1c, *μ*_FE_ vs. normalized *V*_G_ was plotted based on the following equations:


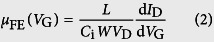



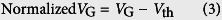


where *V*_D_, *W*, and *L* are drain voltage in the linear region (here, *V*_D_ = 1 V), and channel width and length, respectively. The mobility degradation starting at a certain normalized *V*_G_ value (normalized *Vμ*_FE_) is due to scattering by surface roughness and by Coulombic interactions with fixed and/or trapped charges in the gate insulator^23^,^24^. The normalized *Vμ*_FE_ for the high defect TFT is the smallest with a value of 2.3 V, as listed in Table 1. This indicates that a relatively small amount of electrons is accumulated by a small gate voltage and soon after that, the accumulated electrons begin to be trapped at the interface trap sites and/or injected into the gate insulator. Then, they are captured by trap sites in the gate insulator near the interface, causing Coulombic interactions with channel electrons. This charge trapping/injection starting at a low voltage results in a large hysteresis, as shown in Fig. 1a.

In fact, hysteresis has a Janus-faced property. In non-volatile memory devices, permanent hysteresis where the shifted threshold voltage hardly recovers over time can be used as a kind of memory effect and can be reinforced by using ferroelectric gate insulators^25^,^26^, nanoparticle-embedded gate insulators^27^,^28^, or electric double layers^29^,^30^. On the other hand, the volatile (dynamic) hysteresis of TFTs has been regarded as one of the major problems in display applications since it leads to a threshold voltage shift in the positive direction like a positive bias stress (PBS) and thus, unstable operation of the displays. Therefore, less hysteresis or hysteresis-free TFT devices have been one of the main issues in commercializing TFT technology. However, hysteresis-related defects (interface trap sites and/or trap sites in the gate insulator near the interface) can be a key point for realizing a high S/N ratio and fast recovery in sensor applications, as will be demonstrated in the next section.

### Fast recovery and high S/N ratio in low voltage-driven oxide phototransistors with high hysteresis-related defects

**Figure 2:**
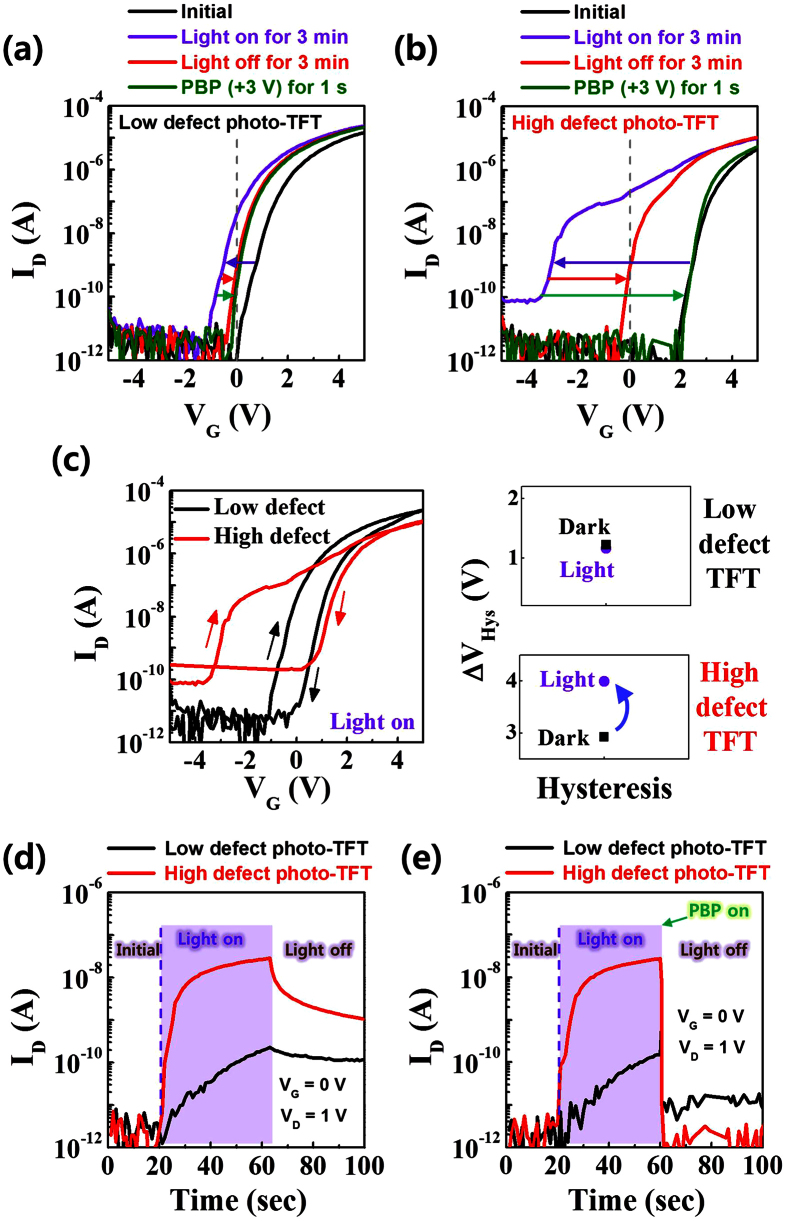
Transfer characteristics of the (**a**) low defect and (**b**) high defect photo-TFTs in four measurement conditions: i) when a positive gate bias pulse voltage (PBP) of 3 V was applied for about 1 s, ii) light with a wavelength of 400 nm was illuminated for 3 min, iii) the light was off for 3 min, and iv) the PBP was applied again. (**c**) Hysteresis characteristics of low defect and high defect TFTs under light illumination. The drain current of the low defect and high defect photo-TFTs under pulsed illumination (**d**) without the PBP and (**e**) with the PBP.

Figure 2a,b show the variations of the transfer characteristics of the low defect and high defect TFTs, respectively, at the initial state, under 400 nm monochromatic light illumination with a power density of 0.1 mW cm^−2^ (3 min), in the light-off state, and with a positive gate bias pulse voltage (PBP) of 3 V for about 1 s. To enhance the S/N ratio, the transfer curves at the initial state were obtained after applying the PBP to the devices in the dark because the PBP also causes negative charges (electrons) trapped at the interface trap sites or injected into the trap sites in the gate insulator near the interface, like the gate sweep in the hysteresis measurements. When 400 nm monochromatic light with a power density of 0.1 mW cm^−2^ was illuminated on the devices for 3 min, the on-currents of both TFTs increased and the transfer curves shifted to the negative direction. This can be attributed to the photo-induced detrapped/ejected electrons from the interface as well as the photo-excited electrons from deep subgap states such as neutral oxygen vacancies (V_O_)^31^,^32^, and/or band tail states^32^. More specifically, the *V*_th_ for the low defect photo-TFT shifted by −1.2 V while the Δ*V*_th_ for the high defect TFT was −5.5 V and unlike the low defect photo-TFT, the off-current increased by about 2 orders of magnitude. The large Δ*V*_th_ and the increment of the off-current in the high defect TFT may be attributed to a relatively large amount of neutral oxygen vacancies (V_O_) in the bulk semiconductor. This speculation is reasonable since the previous report about the effect of deposition power density on IGZO films has demonstrated that bulk defects in the IGZO films increase with increasing deposition power density^33^. Furthermore, the bandgap (E_G_) of IGZO films decreases with increasing deposition power density as shown in Supplementary Figure S1. This indicates that the valence band tail states can significantly contribute to the photo-activated electrons and hence, the off-current will increase with increasing deposition power density, as observed in Fig. 2b. Figure 2c and Supplementary Figure S2 show the hysteresis characteristics of the low defect and high defect TFTs with/without light illumination. The Δ*V*_Hys_ of the high defect TFT under light illumination is increased by 1.1 V as compared to that in the dark, while the Δ*V*_Hys_ of the low defect TFT under light illumination is similar with that in the dark. Kuk *et al.* ascribed the light-induced hysteresis to the photo-ionized oxygen vacancies (V_O_^2+^) at the interface because the V_O_^2+^ in bulk semiconductor near the interface can diffuse and temporarily trapped to the interface due to applied negative gate bias during the forward sweep^34^. Thus, the relatively large change in Δ*V*_Hys_ of the high defect TFT is due to a relatively large amount of V_O_ in bulk semiconductor near the interface.

As for the ambient effect when the oxide channel-layer is illuminated, the desorption of the oxygen-related ions (O_2_^−^, O^−^, etc.) from the back-channel surface results in the enhancement of the negative *V*_th_ shift, owing to the release of free electrons^35^. Chen *et al.* investigated the unpassivated IGZO TFTs under light illumination in both vacuum and O_2_ ambience^36^. They discovered that the *V*_th_ shift in vacuum is larger than that in ambient O_2_ and that *V*_th_ recovery in vacuum is slower than that in ambient O_2_. This is because desorption of the oxygen-related ions in vacuum occurs faster than in ambient O_2_. Thus, they concluded that the dominant mechanism for the un-passivated device under light illumination is photo-desorption of the oxygen-related ions, whereas the *V*_th_ recovery is due to re-adsorption of O_2_ from outer ambience. Therefore, in order to investigate the ambient effect under light illumination, we also measured the transfer characteristics of the TFTs under light illumination in vacuum and O_2_ ambience. As shown in Supplementary Figure S3, the *V*_th_ shift in vacuum is similar to that in ambient O_2_ unlike Chen’s results. In addition, the *V*_th_ recovery in vacuum and ambient O_2_ after the light switch-off (1000 s later) is similar. This independence of light illumination and recovery behavior on ambience indicates that the ambient effect is minor in the photo-responses of our TFT devices. This conclusion is reasonable because AFM measurements revealed that the surface roughness is not dependent on deposition power density but is similar with each other as seen in Supplementary Figure S4.

The shifted transfer curves of both of the TFTs under light illumination slowly recovered after the light was turned off. The slow recovery of oxide semiconductors is attributed to the outward relaxation of metal cations around oxygen vacancies when illuminated^13^,^14^. In addition, there are energy barriers to recover from the metastable state to the original state^13^. This recovery behavior was monitored by time-current measurements, where the drain current (*I*_D_) at *V*_G_ = 0 V and *V*_D_ = 1 V was measured over time. During the measurement, 400 nm monochromatic light with a power density of 0.1 mW cm^−2^ was illuminated on the devices for about 40 s and after the light turned off, the light-off state current was monitored. Figure 2d clearly shows the persistent photocurrent (PPC) which flows in the TFT channels under the light-off state. The PPC can be quickly removed by a PBP of 3 V for about 1 s, as shown in Fig. 2e. Note that the light-off state current of the high defect photo-TFT is as low as 1 pA while that of the low defect photo-TFT is an order of magnitude higher (about 10 pA). This can be easily understood considering the transfer curves of the photo-TFT after applying the PBP, as presented in Fig. 2a,b. The Δ*V*_th_ between the light-on state and light-off state with the PBP is relatively small with a value of 0.6 V for the low defect photo-TFT (see Fig. 2a) and hence, the *I*_D_ at *V*_G_ = 0 V is still at a level of 10 pA. Meanwhile, it is large (4.9 V) for the high defect photo-TFT and the *I*_D_ at *V*_G_ = 0 V is at the original off-current level (see Fig. 2b). This difference of the effect of the PBP is due to the difference of the deposition power density-derived hysteresis (interface trap sites and/or trap sites in the gate insulator near the interface). In other words, large charge trapping/injection results in the effective elimination of the PPC.

**Figure 3:**
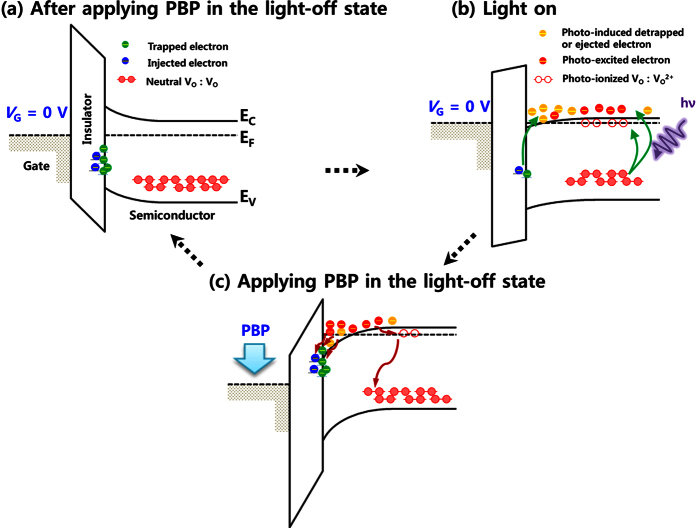
Schematic illustration of the mechanisms of the fast recovery and low light-off state current. (**a**) After applying the PBP in the darkness, the trapped or injected electrons near the interface deplete carrier electrons and make upward band-bending. (**b**) When the light is switched on, the electrons near the interface are detrapped or ejected from the interface and simultaneously, subgap states in the bulk semiconductor are excited and eject the photo-excited electrons. Consequently, the Fermi level (E_F_) increases and *V*_th_ is negatively shifted. (**c**) When the light is switched off and the PBP is applied, the detrapped/ejected electrons and the photo-excited electrons are trapped/injected again and recombined with the excited subgap states.

Figure 3 illustrates the mechanisms of the fast recovery and low light-off state current. After applying the PBP, the trapped or injected electrons near the interface deplete carrier electrons and produce upward band-bending. This results in the low off-current at a level of 1 pA (see Fig. 3a). When the light is switched on, the trapped or injected electrons near the interface are detrapped or ejected from the interface and simultaneously, oxygen vacancies and tail states in the bulk semiconductor are excited and eject the photo-excited electrons. Consequently, the Fermi level (E_F_) increases and *V*_th_ is negatively shifted (see Fig. 3b). When the light is switched off and the PBP is applied, the detrapped/ejected electrons and the photo-excited electrons are trapped/injected again and recombined with oxygen vacancies or tail states (see Fig. 3c). This leads to the upward band-bending again.

**Figure 4:**
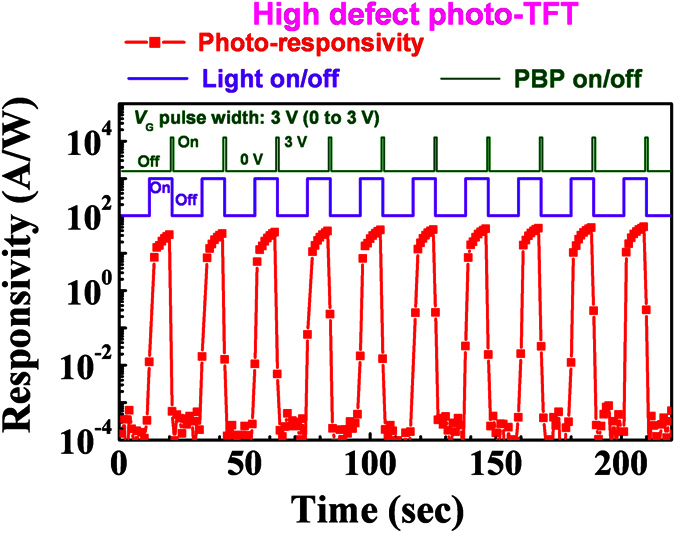
Repeated sensing property of the high defect photo-TFT showing high sensitivity and fast recovery. The operating voltages are *V*_G_ = 0 V, *V*_D_ = 1 V, and PBP = 3 V. Light was switched off for about 20 s and switched on for about 20 s. The PBP was applied for about 1 s, right after the light was switched off.

**Figure 5:**
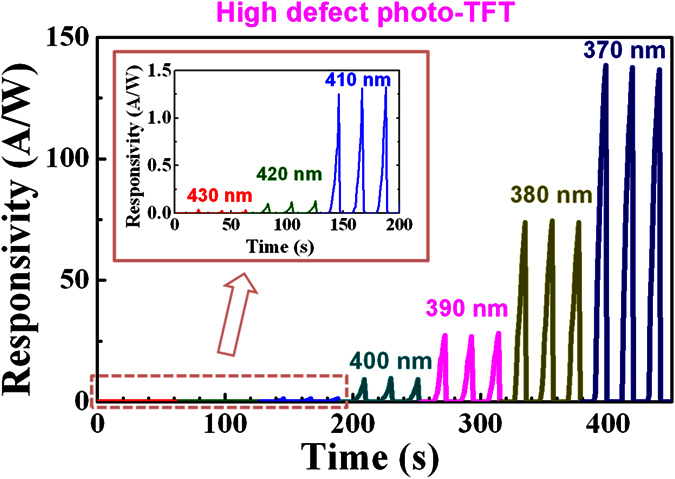
Responsivity of the high defect photo-TFT under light with various wavelengths.

We confirmed that the high defect photo-TFT has a stable and repeatable sensing property. Figure 4 shows that the high defect photo-TFT operated well repeatedly, having a high responsivity, fast recovery time, and low light-off state current. Note that the operating voltages are *V*_G_ = 0 V and *V*_D_ = 1 V, and PBP = 3 V. These are much lower than those obtained in previous reports^13^,^14^. Some groups have investigated photocurrent in oxide semiconductor using stretched exponential analysis and gate-pulse spectroscopy analysis^37^,^38^, and suggested that the photocurrent and PPC were due to metastable oxygen defect ionization. In our work, however, the top-gat IGZO TFTs exhibited a smaller V_th_ shift (ΔV_th_ = ~0.3 V) under illumination stress compared to the bottom-gate photo-TFT, although the same growth conditions were used for the deposition of IGZO channels. In addition, the high defect photo-TFTs demonstrated good repeatable photo-sensing characteristics during long-term photo-sensing tests without electrical degradation, as shown in Supplementary Figure S6. Thus, although the oxygen defects ionization model is partially responsible for the photo-instability of our phototransistors, carrier trapping/detrapping seems to be the primary source of the photocurrent in high defect photo-TFTs. Finally, we measured the responsivity of the high defect photo-TFT illuminated with various wavelengths ranging from UV (370 nm, 3.35 eV) to blue (430 nm, 2.88 eV). Here, the responsivity (*R*) at a given wavelength (*λ*) was calculated by the following equation^13^:


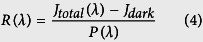


where *J*_*total*_ is the total current density when the device is illuminated, *J*_*dark*_ is the current density in the darkness, and *P* is the power density of the light. As seen in Fig. 5, the responsivity increases monotonously with decreasing wavelength by 10 nm. This is because deeper defect states are excited as *λ* decreases and hence, the amount of the excited subgap states increases. It is noticeable that our photo-TFTs (bottom-gate IGZO photo-TFTs with ALD-derived Al_2_O_3_ gate insulators) adopt a simple channel structure (one-layer) and use hysteresis-related defects for a high S/N ratio and fast recovery that are induced by an easy process (control of the deposition power density). These findings make our study different from previous studies where complex channel (double- or tri-layer) or dual-gate structures were adopted and the elimination of the PPC for fast recovery was explained only by the recombination mechanism between oxygen vacancies and excess electrons^13^,^14^,^15^.

## Discussion

We demonstrated that the surface of the ALD-derived Al_2_O_3_ gate insulator was damaged by ion bombardment during the IGZO sputter deposition process. Such damage resulted in the hysteresis behavior of the bottom-gate IGZO TFTs and the degree of the hysteresis increased with increasing deposition power density. This indicates that we can easily control the amount of the interface trap sites and/or trap sites in the gate insulator near the interface. We also showed that such hysteresis-related defects were useful for photo-sensor applications. The photo-TFT with large hysteresis-related defects had a low light-off state current (high S/N ratio) and fast recovery even though the operation voltages were low (*V*_G_ = 0 V, *V*_D_ = 1 V, and PBP = 3 V). Furthermore, through the easy process for high S/N ratio and fast recovery at low operation voltages (control of the deposition power density), we obtained a high sensitivity since the bulk defects that can be excited and eject electrons by illumination also increased with increasing deposition power density. Also, this photo-TFT showed clearly distinguishable wavelength dependence of photo responsivity.

## Methods

### TFT Fabrication

The staggered bottom-gate and top-gate type IGZO TFTs were fabricated using 100 nm thick Al_2_O_3_ gate insulators on glass substrates. Al_2_O_3_ dielectric layers were deposited at 150 °C by atomic layer deposition (ALD) with trimethylaluminium (TMA) and water (H_2_O) precursors. 100 nm thick molybdenum (Mo) was deposited by direct current magnetron sputtering and used as the gate and source/drain (S/D) electrodes. All electrodes were patterned by a lift-off process. The channel length (*L*) and width (*W*) were 50 and 500 μm, respectively. The IGZO channel-layers were deposited by RF magnetron sputtering using a 4-inch IGZO target (In:Ga:Zn atomic ratio of 2:1:2). Different deposition powers of 0.62 and 1.85 W cm^−2^ were applied to the target and the deposition time for each deposition power was controlled to allow the thickness of the channel-layers to be about 60 nm. The channel-layers and gate insulators were defined by a conventional photolithography and wet-etching process. All devices were thermally annealed at 350 °C in air for 1 h.

### Characterizations of Thin Films

The thicknesses of the channel layers, gate insulators, and electrodes were measured by an alpha-step surface profilometer (XP-100, Ambios Technology, Inc.). The transmittance of the IGZO films was measured by a UV-VIS spectrophotometer (UV-1800, Shimadzu Corp.).

### TFT Measurements

The current-voltage (I-V) measurements were carried out in air using an HP-4145B semiconductor parameter analyzer. A 150 W Xe arc lamp (LS-150, ABET Technologies Inc.) was used as the light source and the light wavelengths with a full-width-at-half-maximum of about 20 nm were selected by a monochromator (Monora 200, Dongwoo Optron Co., Ltd.). The optical power of the monochromatic light was measured using a UV-enhanced Si detector.

## Additional Information

**How to cite this article**: Yun, M. G. *et al.* Low voltage-driven oxide phototransistors with fast recovery, high signal-to-noise ratio, and high responsivity fabricated via a simple defect-generating process. *Sci. Rep.* **6**, 31991; doi: 10.1038/srep31991 (2016).

## Supplementary Material

Supplementary Information

## Figures and Tables

**Table 1:** Summary of the extracted parameters of the IGZO TFTs.

TFT	*V*_th_ (V)	Δ*V*_Hys_ (V)	SS (V dec^−1^)	*μ*_FE_ (cm^2^ V^−1^ s^−1^)	Normalized *Vμ*_FE_ (V)
Low defect TFT	0.1	1.2	0.24	10.3	5.5
High defect TFT	0.0	2.9	0.20	3.0	2.3
Top-gate TFT	−6.2	0.3	0.28	11.4	7.4
