# Supplementary material for: Low voltage-driven oxide phototransistors with fast recovery, high signal-to-noise ratio, and high responsivity fabricated via a simple defect-generating process
Source: Sci Rep. 2016 Aug 24;6:31991. doi: 10.1038/srep31991 (PMC4995484; doi:10.1038/srep31991)
Supplement: Supplementary Information [file srep31991-s1.pdf]

Supplementary Information

Low voltage-driven oxide phototransistors with fast recovery, high signal-to-noise ratio, and high responsivity fabricated via a simple defect-generating process

Myeong Gu Yun[1‡], Ye Kyun Kim[1‡], Cheol Hyoun Ahn[1‡], Sung Woon Cho[1], Won Jun Kang[1], Hyung Koun Cho[1], and Yong-Hoon Kim[1,2]*

[1]School of Advanced Materials Science and Engineering, Sungkyunkwan University, 2066 Seobu-ro, Jangan-gu, Suwon, Gyeonggi-do, 16419, Republic of Korea

[2]SKKU Advanced Institute of Nanotechnology (SAINT), Sungkyunkwan University, 2066 Seobu-ro, Jangan-gu, Suwon, Gyeonggi-do, 16419, Republic of Korea

*E-mail: chohk@skku.edu

[‡]These authors contributed equally to this work.

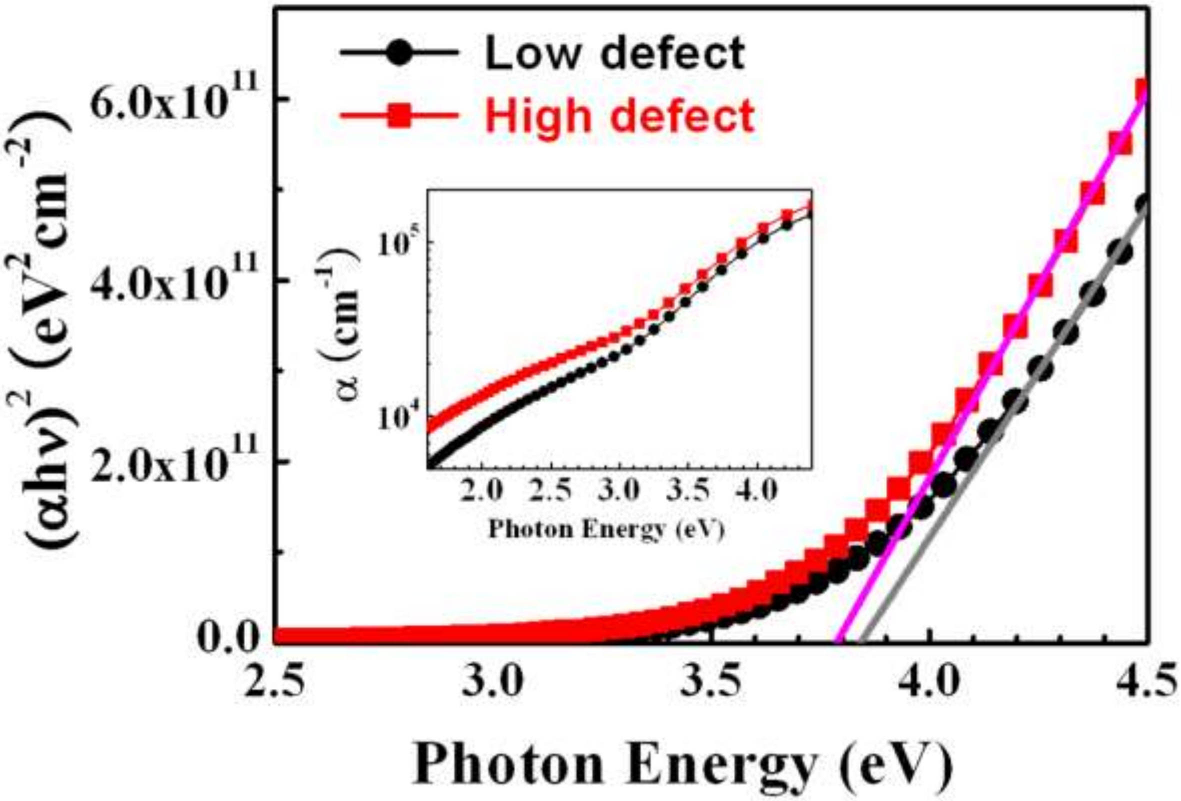

Figure S1. Optical properties of the low and high defect IGZO films on glass substrates, which is obtained from the transmittance data of the films. From Tauc plots; $(\alpha h\nu)^2$ as a function of the photon energy, $h\nu$, where α is the absorption coefficient, h the Planck's constant, and ν the photon frequency, the bandgaps of the low and high defect IGZO films were estimated to be 3.84 and 3.79 eV, respectively. The inset shows the absorption coefficients of the low and high defect IGZO films.

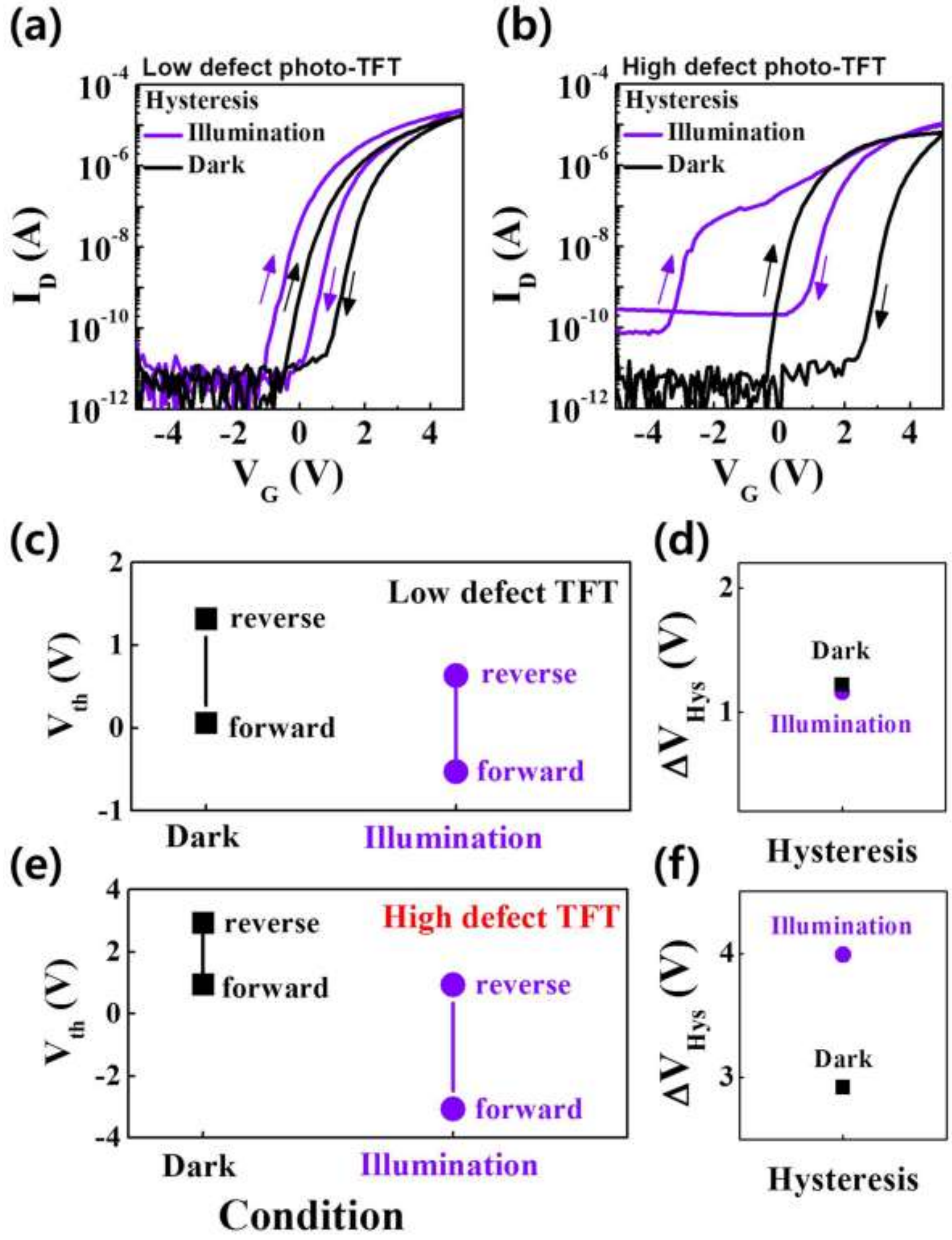

Figure S2. Hysteresis loops of the (a) low defect and (b) high defect TFTs with and without light illumination. (c), (e) Measured V_{th} and (d), (f) calculated ΔV_{Hys} are also presented.

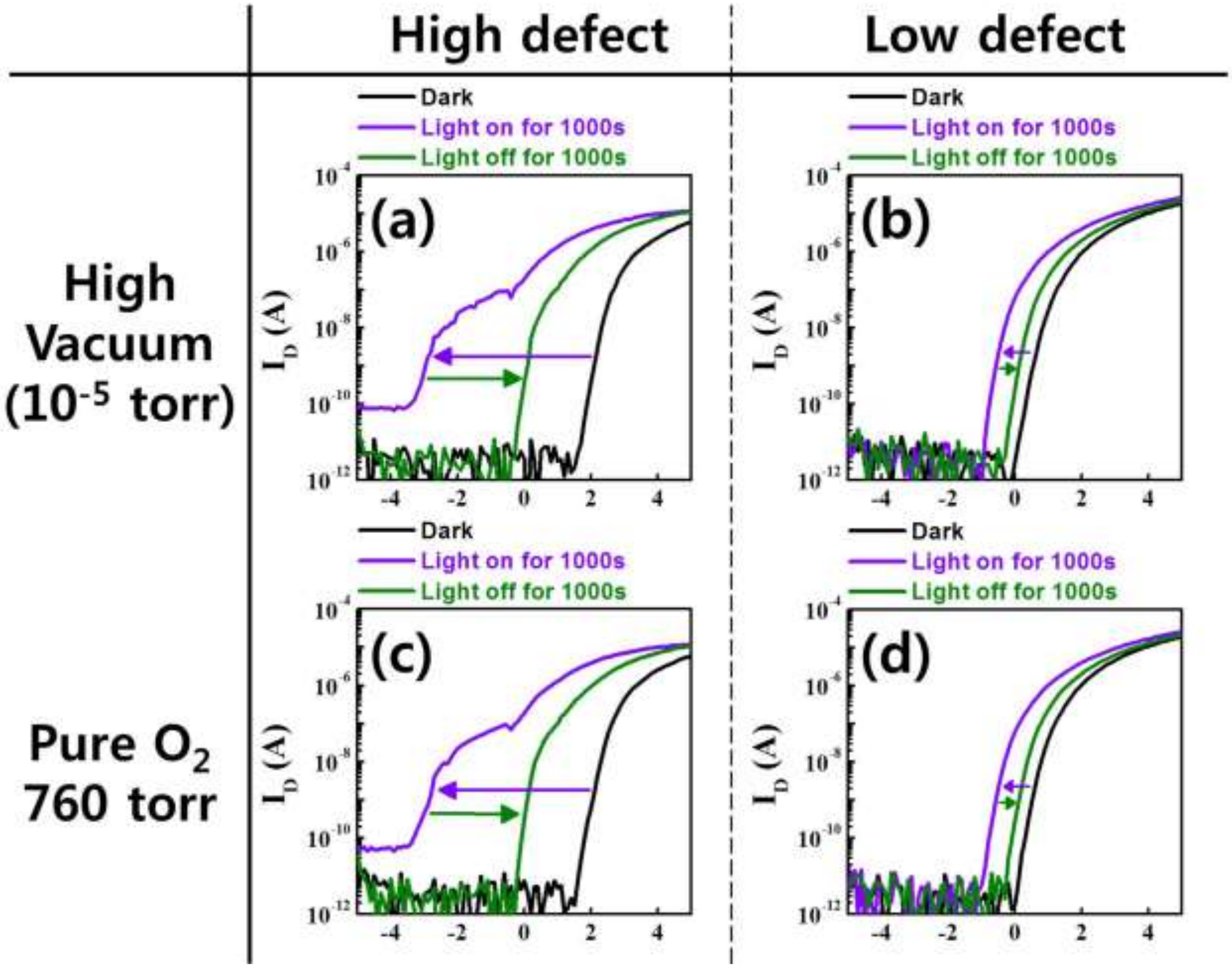

Figure S3. Transfer characteristics of the (a) high defect and (b) low defect TFTs in vacuum (10^{-5} torr) under three measurement conditions: i) in the darkness, ii) under light illumination for 1000 s, and iii) in the light-off state for 1000 s. Transfer characteristics of the (c) high defect and (d) low defect TFTs in ambient O_2 (760 torr) under the same measurement conditions.

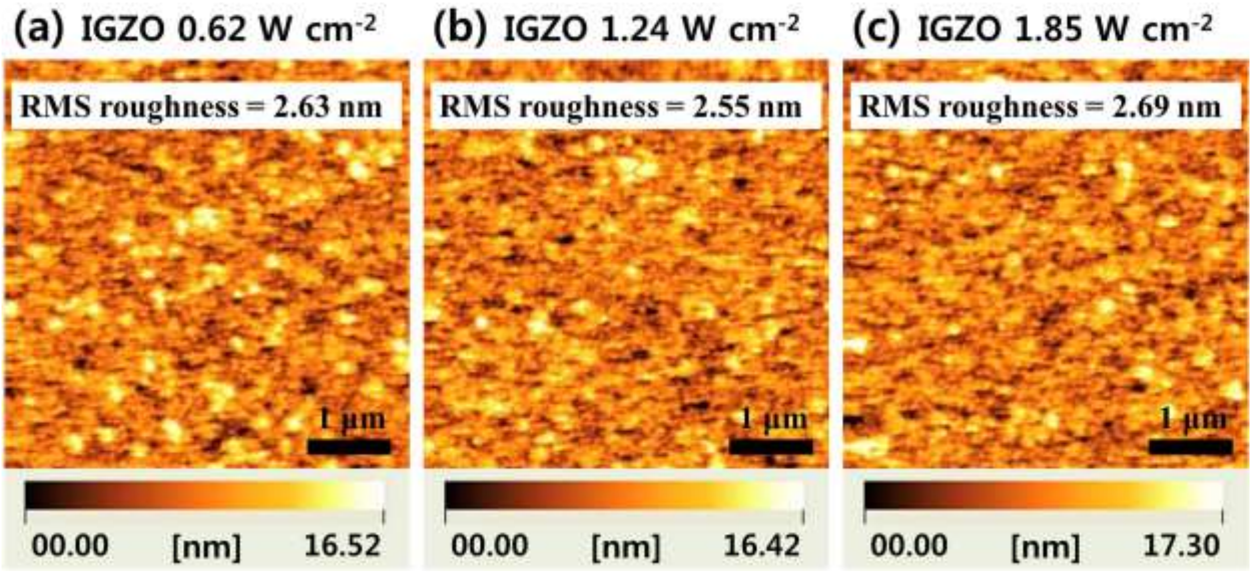

Figure S4. AFM images and surface roughness of IGZO films deposited on Al_2O_3/Mo/Glass structures with various deposition power densities.

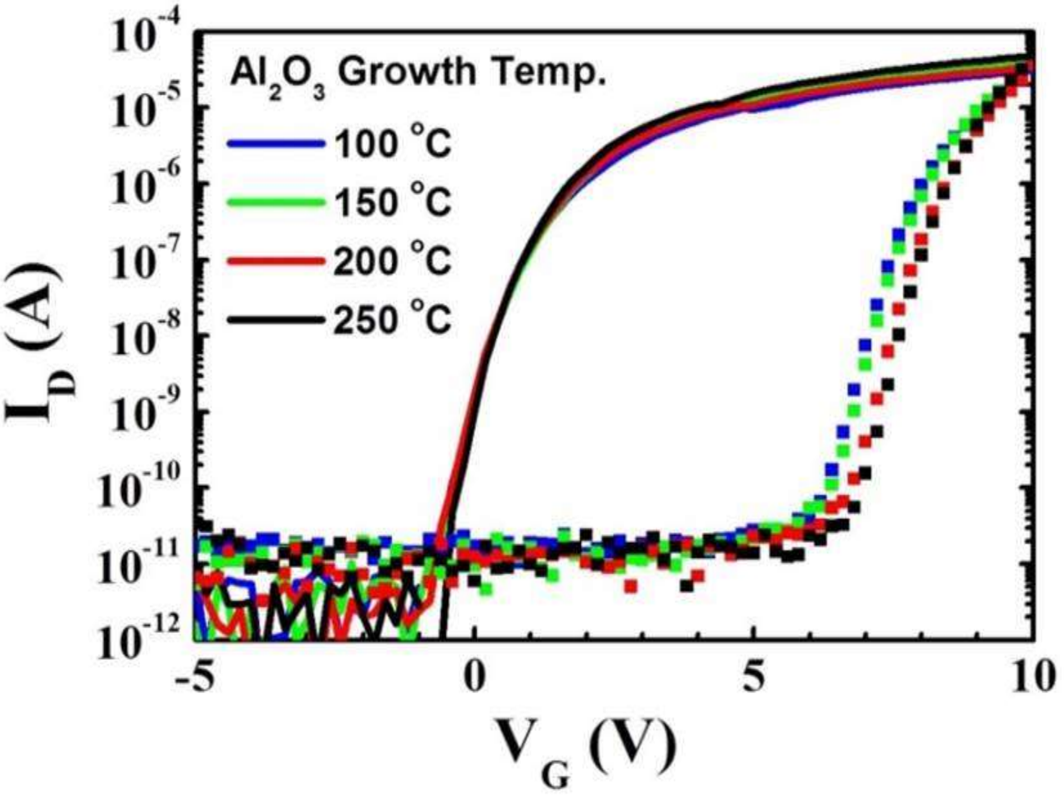

Figure S5. Hysteresis characteristics of the bottom-gate IGZO TFTs with Al_2O_3 dielectric layers prepared at different growth temperatures.

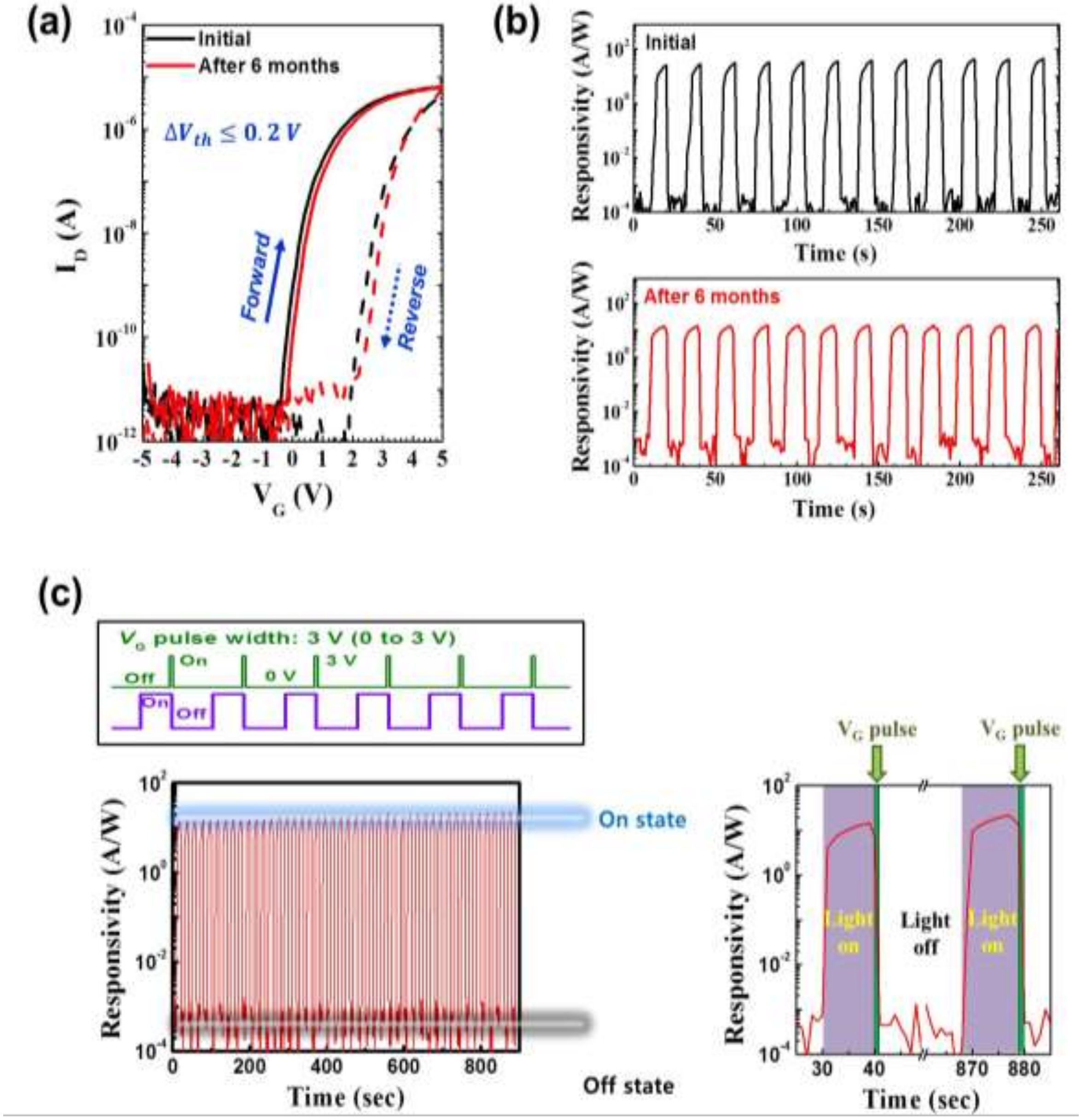

Figure S6. (a) Transfer curves and (b) photo-sensing curves of a high defect photo-TFTs showing reproducibility of performance. (c) Long-term repeated photo-sensing properties of a high defect photo-TFTs.

Table S1. Hysteresis-related defect density (N_T) of the TFTs depending on deposition power density and the TFT structure.

TFT			ΔV_{Hys} (V)	N_T (cm^{-2})
**Structure**	**Deposition power density ($W\ cm^{-2}$)**	**Naming**		
**Bottom-gate**	0.62	**Low defect TFT**	1.2	6.2×10^{11}
**Bottom-gate**	1.85	**High defect TFT**	2.9	1.5×10^{12}
**Top-gate**	1.85	**Top-gate TFT**	0.3	1.5×10^{11}
